# Global Status of Vegetable Soybean

**DOI:** 10.3390/plants12030609

**Published:** 2023-01-30

**Authors:** Ramakrishnan M. Nair, Venkata Naresh Boddepalli, Miao-Rong Yan, Vineet Kumar, Balwinder Gill, Rabi S. Pan, Chansen Wang, Glen L. Hartman, Renan Silva e Souza, Prakit Somta

**Affiliations:** 1 World Vegetable Center South Asia, ICRISAT Campus, Hyderabad 502324, Telangana, India; 2 Department of Agronomy, Iowa State University, Ames, IA 50011, USA; 3 World Vegetable Center, Shanhua, Tainan 74199, Taiwan; 4 ICAR-Indian Institute of Soybean Research, Khandwa Road, Indore 452001, Madhya Pradesh, India; 5 Department of Plant Breeding & Genetics, Punjab Agricultural University, Ludhiana 141004, Punjab, India; 6 ICAR Research Complex for Eastern Region, Farming System Research Centre for Hill and Plateau Region, Plandu, Ranchi 834010, Jharkhand, India; 7 Department of Agronomy, National Chung Hsing University, South District, Taichung 40227, Taiwan; 8 USDA-ARS, Soybean/Maize Germplasm, Pathology, and Genetics Research Unit, 70 National Soybean Res Center, University of Illinois, W. Peabody Dr., Urbana, IL 1101, USA; 9 Institute of Plant Breeding Genetics and Genomics, University of Georgia, Athens, GA 30602, USA; 10 Department of Agronomy, Faculty of Agriculture Kamphaeng Saen, Kasetsart University, Nakhon Pathom 73140, Thailand

**Keywords:** *edamame*, vegetable soybean, nutritional quality, breeding, biotic stress, abiotic stress

## Abstract

Vegetable soybean, popularly known as *edamame* in Japan and *mao dou* in China is a specialty soybean. Green pods with physiologically mature beans are harvested, and whole pods or shelled beans are used as a fresh or frozen vegetable. Vegetable soybeans are prepared in diverse ways, and they are highly nutritious, with excellent taste properties. Unlike grain soybeans, it is perishable. In this review, the chronological progression of area, production, export, import, and expansion of vegetable soybeans and potential for further expansion is discussed. Available information on current ongoing research and development activities in various countries around the world are presented, and their relevance is discussed. At present, the production and consumption of vegetable soybeans are mainly in East and Southeast Asia, with Japan as the largest importing country that dictates the global market. However, interest and trend in cultivation of this crop in other regions has increased significantly. Lack of germplasm or suitable varieties is a major constraint in vegetable soybean production and expansion in countries outside East and Southeast Asia. Most of the vegetable soybean varieties are genetically related and are susceptible to biotic and abiotic stresses. Extensive research and breeding of vegetable soybeans are still restricted in a few countries such as China, Japan, Taiwan and the USA. The need for focused research and development activities with concern for the environment, farmers’ and processors’ profit, consumers’ preference, quality, and nutrition are emphasized.

## 1. Introduction

**Figure 1:**
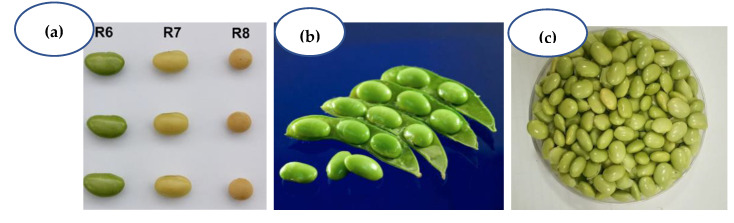
(**a**) Vegetable soybeans at the R6, R7, and R8 stages; (**b**) pods harvested at the ideal stage (R6); (**c**) beans in R6 ready for consumption.

Vegetable soybean (*Glycine max* (L.) Merrill) is a highly nutritious leguminous vegetable crop rich in protein (13% at R6 stage), iron, and calcium [1], nutrients that are an essential part of the human diet to combat chronic malnutrition in the world. Vegetable soybeans are also known as ‘*edamame’* (Japanese), ‘*mao dou’* (Chinese), ‘*Poot kong’* (Korean), beer beans, sweet beans, and green soybeans (in other parts of the world). Large seed size (>60–75 g/100 seeds fresh weight), sweeter in taste, and without beany flavor are the major traits of vegetable soybeans which distinguish them from grain soybeans. Pods harvested at the R6 growth stage (when the pods are still green, and the beans in the pods are 80% mature) can be sold in markets as fresh, frozen, and canned products [2] (Figure 1). Green beans can be served within pods as a snack or appetizer after boiling in salt water, or the shelled green beans can be sautéed with meats or other vegetables and used in soups, curries, and salads. Vegetable soybeans can be used instead of garden peas (*Pisum sativum*) [3] or lima beans (*Phaseolus lunatus*) in any recipe [4]. With its low input requirement, short duration (65–75 days to harvest), and soil nitrogen fixing ability, vegetable soybeans fit in a wide range of cropping systems [5]. After stripping the pods, green plant stalks with leaves can be fed to livestock [6] or incorporated into soil as green manure to enrich soil health [7].

While vegetable soybeans are not yet well known by people in Europe, Africa, and South and West Asia, it is popularly produced and consumed by East and Southeast Asian people and plays an important role in diets and agriculture in those regions. However, due to its high nutritional value and excellent taste properties, interest in the cultivation of this crop has increased significantly in several countries outside East and Southeast Asia during the last decade. Since vegetable soybeans can be produced in a short period of time, nearly all the parts of vegetable soybeans are utilizable, and the ability of the vegetable soybeans in association with *Rhizobia* to fix atmospheric nitrogen to the soil, this legume crop fits well with several cropping systems and can improve sustainability in agriculture. For example, in Thailand, farmers generally grow vegetable soybeans as a cash crop after major crops such as rice and maize, and after harvesting the pods, the plant parts are used to produce compost and to feed dairy animals. Although the production, uses, and export of vegetable soybeans in major producing countries appear to be promising, there are still several constraints and limitations in the production of this crop. For example, (i) most of the varieties grown for export to the major imported country, Japan, are closely genetically related and are susceptible to insects and diseases, resulting in extensive use of pesticides in production, (ii) the lack of germplasm limits developing varieties adapted to different regions or possessing eating qualities for domestic/local preferences, and (iii) the production of vegetable soybeans requires a high amount of water, limiting the expansion of the production to certain areas. So, there are needs for focused research and development activities to solve the limitations of vegetable soybean production. To the best of our knowledge, there is no detailed review on vegetable soybeans. In this paper, we review and discuss the global status of vegetable soybean production and research on this special legume crop.

## 2. Nutritional Considerations

Vegetable soybeans are an excellent source of digestible proteins (13% in the R6 stage; Figure 1), carbohydrates (20–30%) lipids (19%), essential fatty acids, phosphorous, iron, calcium, zinc, thiamine, riboflavin, vitamin E (tocopherol), dietary fiber (16%), and sugar [8,9,10,11]. Both cysteine and methionine content in protein is slightly lower in vegetable soybeans than grain soybeans. Trypsin inhibitor activity is lower in vegetable soybeans than their grain counterpart. The major sugar content in beans is in the form of sucrose (74%), fructose (3%), and glucose (3%) [12]. Sucrose contributes 72% of total soluble sugars (6 g to 12 g/100 g on fresh weight basis) at the edible stage of vegetable soybeans [11,13,14]. A significant positive correlation has been reported between the eating quality score and sucrose content in 30 vegetable soybean varieties grown in China [15]. In general, vegetable soybeans are sweeter in taste at the R6 stage compared to grain soybeans which have an oily taste. Genetic differences in sucrose content have been observed among vegetable soybeans. Vegetable soybean lines AGS406 and AGS447 were found to be sweeter than the grain soybean variety (JS-335) in organoleptic tests conducted in Dharwad, India [16]. High levels of essential amino acids (glutamic acid, alanine, histidine, and asparagine) in the beans were also reported by Maruthi et al. [17] and Guo et al. [18], and these amino acids also play a vital role in flavor and sweetness of vegetable soybeans. Vegetable soybeans contain natural isoflavones (48.95 mg/100 g), which can lower the risk of cancer [19], cardiovascular diseases, and osteoporosis [20]. The isoflavones in vegetable soybeans are in the form of glycosides: genistin (β-glucosides), daidzin (acetyl-β-glucosides), glycitin (malonyl-β-glucosides) [21], and aglucones [9]. Poultry feed enriched with vegetable soybean isoflavones (3 g/kg of feed) has enhanced the carcass quality of broiler chickens by reducing the fat percentage and level of cholesterol [22]. In addition, unmarketable pods and beans from the processing industry have been fermented to make vegetable soybean chips for use as poultry feed. Fermented vegetable soybean meal enhances immunity in black-boned chickens [23] and also improves muscle weight [24]. Fermented soymilk from vegetable soybeans was reported to be a good food matrix to deliver probiotic bacteria, as well as a soy product with a lower content of non-digestible oligosaccharides [25]. Fermentation reduces the contents of stachyose and raffinose in soymilk. The soy components that have stimulated the most research interest are isoflavones with estrogenic properties [26]. The low content of bioactive compounds in second generation soy foods and moderate amounts in traditional soy preparations offer modest health benefits with very limited risk for potential adverse health effects [27].

## 3. Global Area, Production, and Productivity

Awareness about the health and nutritional benefits of vegetable soybeans and demand from both domestic and international markets have caused an increase in acreage of the crop in countries in Asia (28–40% in the last fifteen years), the Americas, Europe [28], and sub-Saharan Africa [29]. Currently, the leading vegetable soybean producers are China, Japan, and Taiwan (Table 1), while the major consumers are China, Japan, Korea, the USA, Taiwan, Thailand, and Europe. China is the leading producer with 90% of the total area (about 400,000 ha) and production (2,000,000 tons per year) [7], followed by Japan (13,000 ha; 66,100 tons) and Taiwan (9180 ha; 84,490 tons) [28,30,31]. Zhejiang, Fujian Anhui, Shanxi, and Jiangsu provinces in the coastal area of Eastern China are major vegetable soybean producing and processing areas [28]. In Japan, major production regions include Hokkaido, Akita, Yamagata, Gunma, and Chiba, with about 1200–1570 ha planted in each region in 2019 [32]. In Thailand, total planting area is about 4000 ha with total yield of 25,000 t [33]. The major production areas for export are in the northern region include Chiang Rai, Chiang Mai, Payao, and Uthai Thani, while the main production areas for domestic use are in the lower central regions, including Kanchanaburi, Lopburi, Saraburi, Sing Buri, Ang Thong, and Ayutthaya. The production of vegetable soybeans is year-round, but the production for export can be made from two crops. In Indonesia, vegetable soybeans are produced primarily in Jember in East Java. Over a period of 20 years, the total harvested area of vegetable soybeans has increased from 30.5 ha (1994–1998) to 1417 ha (2017) in Indonesia due to its demand for export [34]. In the USA, vegetable soybeans have been grown commercially and processed in Arkansas, where a dedicated processing plant was built to process vegetable soybeans [35,36]. Other growers in the USA are SunRich in Minnesota and Cascadian Farms in Washington State [37]. Vegetable soybeans have become the second largest soyfood consumed in the USA at 25,000 to 30,000 t annually [38]. Canada continues to export food grade soybeans, including vegetable soybeans to the Asian market (https://soycanada.ca/industry/industry-overview/ (accessed on 1 February 2022)). A study conducted in Latvia and Norway [39] showed the potential for successful cultivation of vegetable soybeans at higher latitudes, such as the Nordic–Baltic region in North Europe, with yield levels comparable to other regions of the world.

Japan is the largest vegetable soybean importer in the world and the demand was about 135,000 t during 2020 [32]. Taiwan, China, Thailand, and Indonesia are the four major vegetable soybean exporters to the Japan market that imported about 77,600 and 71,100 tons with a value of USD 161.0 and 199.2 million in 2019 and 2020, respectively [32]. The decline in imports in 2020 was possibly due to the outbreak of the COVID-19 pandemic. In fact, compared to 1987, current production in Japan is only slightly changed, while the imports increased nearly twofold. In Thailand, about 70% of vegetable soybeans are exported to Japan as frozen pods and frozen shelled beans. However, domestic consumption increased in recent years due to consumer perception of the health benefits of vegetable soybeans. Vegetable soybeans sold in markets across the USA are mainly imported from Canada, China, Guatemala, and Taiwan and are mostly marketed frozen either with or without the shell. At least 70% of the green vegetable soybeans consumed in the USA are imported, mainly from China and Taiwan. Increased demand has resulted in a steady increase in land acreage under vegetable soybeans in the USA [40,41]. An analysis of Indonesia’s export market showed that the Japanese market was too competitive [33]. Indications of near saturation of the Japanese market meant that Indonesia needs to explore other countries, such as the US and Europe, where there is more demand. The need for improving the quality and the quantity of vegetable soybeans produced in Indonesia were highlighted for sustainable growth. In an analysis of the vegetable soybean supply chain in Indonesia, Marimin et al. [42] identified failures to comply with the order quantity and time delivery schedule as major factors that decrease the farmers’ group efficiency.

In Thailand, while grain soybean production has hugely declined over the past 10 years, vegetable soybean production has increased. Although most of the production is for export, domestic consumption has increased significantly. The successful production for export in Thailand is due to a fair and strong contract farming system between companies and farmers. Vegetable soybeans were promoted for domestic consumption and production around the mid-1980s using local grain varieties. Then, vegetable soybean germplasm (mostly Japanese varieties) from WorldVeg was introduced and reselected for both domestic consumption and export. Such varieties are KPS292 (AGS292), No. 75 (Ryokkho 75) and No. 2808. At present, the most popular variety used for production and export is 75A introduced from Taiwan. The variety can be grown year-round with the exception of March and April. A special variety, Chiang Mai 84-2, possessing a pleasant aroma (pandan-like aroma), high yield, and acceptable eating quality has been developed for export for a niche market in Japan [43]. A cultivar with unknown origin, Tharn Kasem, is popularly grown for domestic consumption for consumers who prefer intact pods (no splitting) after boiling, golden yellow pods, and crunchy seeds [44]. In addition, Thai consumers conventionally prefer vegetable soybeans with a creamy and beany taste/aroma. However, another trait that is mainly focused on selection by famers is seed germination/storability. In general, soybean cultivars with large seeds/pods showed field emergence and short-period storability. Thus, the cultivars developed/selected by the farmers are medium seed/pod size.

The production of vegetable soybeans in other regions is still small, even in countries with significant production of grain soybeans such as Brazil and Argentina. Brazil currently is the largest producer of grain soybeans in the world [45], but cultivation and consumption of vegetable soybeans is not common. An evaluation study in Brazil found that JLM010 was the most recommended genotype for the region of Jaboticabal, owing to its better agronomic performance, producing 136.04 g of fresh weight of pods per plant, 92.52 g of fresh weight/100 seeds, and 11.12 t/ha of total immature seeds, with better functional properties. Although the consumption of vegetable soybeans is still small in these countries, since other soybean foods, such as soy sauce, tofu, soybean texturized protein, among others are well-known, accepted, and widely used, there is a great potential for vegetable soybeans.

Gradual progress has been made on vegetable soybean development in Africa. In 2020, two varieties (Chitedze vegetable soybean 1 and Chitedze vegetable soybean 2) were released in Malawi. In South Africa, since the crop was only introduced in 2009, the cropped area is still small [46]. There are plans to increase production and consumption through initiatives of the Edamame Development Programme and Fair Food Company.

## 4. Constraints to Production

### 4.1. Suitable Varieties

A large number of improved breeding lines were developed by the World Vegetable Center (WorldVeg) from which various countries have developed improved varieties and released them for production in their countries [47]. Among the improved lines of the WorldVeg, the line AGS292 which is a selection from Japanese landrace Taisho Shiroge has been used as the main variety for commercial production and export from China and Thailand for more than 15 years. Due to its relative insensitivity to photoperiod and temperature, which contributed to its wide adaptation, it has been widely used as a parent for further varietal development. AGS 292 is of short stature and very early maturing, with a stable average yield. Improved vegetable soybean varieties developed by both the public and the private sector in China, Japan, Taiwan, and the USA have made a significant contribution in the popularization of the crop. Private seed companies primarily from Japan, Taiwan, China, and the USA continue to be the major source of improved vegetable soybean seed commercially available for vegetable soybean growers. Vegetable soybean seeds from public sources are negligible. However, further varietal improvement in biotic and abiotic stresses for specific countries are needed to expand production. Lack of locally adapted varieties of vegetable soybeans was identified as one of the major factors restraining the commercial production of the crop [29,48]. In commercial diversity studies, introduced exotic varieties showed poor adaptability owing to more water requirements [49] and poor seedling emergence in Australia, the USA, and Uganda [50,51]. Introduced varieties from Japan and Korea were found to be prone to pod shattering in the Midwest USA [52], which is a concern for seed production. In Uganda, G10427 was found to be the best genotype to produce a variety with good attributes, especially large seeds, high yield, and adaptability to local conditions [51]. The need for better varieties (plant size and height of the lowest pod) suitable for mechanical harvesting was emphasized to improve harvest efficiency in China [7].

Domestic production of vegetable soybeans in the U.S. was well behind consumer demand, with approximately 70% of U.S.-consumed vegetable soybeans imported each year. A major barrier for growth of the U.S. vegetable soybean industry is an overall lack of varieties with adequate consumer acceptability and adaption to the U.S. climate and environment. In the evaluation in the mid-Atlantic U.S., Lord et al. [53] found that genotypes V16-0524 and R15-10280 gave favourable yield and resilience to native pests compared to the commercial strain, UA-Kirksey, and showed strong potential to increase the availability of varieties that can be used for commercial vegetable soybean production. Recently, seven improved vegetable soybean germplasm lines in a range of maturities with disease and pest resistance were released that will be valuable for breeders in the U.S. for developing large-seeded, disease and pest resistant cultivars [54].

The genetic structure and diversity of 100 vegetable soybean accessions (77 from Mainland China, 13 from Taiwan, 8 from Japan, and 2 from Thailand) were analysed using 53 simple sequence repeat (SSR) markers [28]. A total of 296 alleles were detected with an average of only 5.6 alleles per SSR locus. The germplasms from China, Taiwan, and Japan were highly similar to each other with over 98% similarity. The narrow base of vegetable soybean germplasm has been reported by different authors. Mimura et al. [55] assessed diversity of 130 vegetable soybean accessions, mostly from Japanese seed companies, using 17 SSR markers and found that Japanese vegetable soybeans have a narrower genetic base than the Chinese vegetable soybeans. Kaga et al. [56] analysed genetic diversity of 1603 soybean accessions including 130 Japanese vegetable soybean accessions collected at the national genebank of Japan using 191 single nucleotide polymorphism (SNP) markers. Clustered analysis showed that most of the Japanese landrace vegetable soybeans were clustered into nine sub-clusters within Japanese soybean germplasm. These clusters comprised principally soybeans showing coloured and large seeds. Nonetheless, little is known about Chinese and Korean landrace vegetable soybeans. The need for broadening the genetic base of Japanese vegetable soybeans through proper selection of parents without compromising the quality was emphasized. The sources of germplasm that could be tapped into included Chinese, US, Canadian, and Korean for the above purpose. Zhang et al. [48] reported that Chinese vegetable soybean accessions have a narrow genetic base. The results also indicated that EST-SSRs from grain soybeans have high transferability to vegetable soybeans and would be helpful in taxonomy, molecular breeding, and comparative mapping studies in the future. Single sequence repeat (SSR) markers have been used to examine the genetic diversity in varieties.

### 4.2. Biotic Factors

All of the same diseases and pests that attack grain soybeans [57], also attack vegetable soybeans although their importance may vary somewhat and certainly vary by production regions throughout the world. Most soybean grain breeding programs have focused on incorporating resistance to at least some of the more important diseases and pests, resulting in most modern cultivars carrying genes for resistance to diseases, such as soybean rust, Phytophthora root and stem rot, soybean cyst nematode, and soybean aphids, to name a few. There has been no similar effort to breed vegetable soybeans for disease and pest resistance, and the crop in general is more vulnerable to pathogens and pests.

#### 4.2.1. Insects

Lord et al. [53] reported the prevalence of the soybean aphid (*Aphis glycines*), potato leafhopper (*Empoasca fabae*), Mexican bean beetle (*Epilachna varivestis*), a complex of stink bugs (*Euschistus* spp., *Chinavia hilaris*, and *Halyomorpha halys*), and lepidopteran larvae (*Hypena Scabra*, *Chrysodeixis includens*), that varied from location and year in the mid-Atlantic US. The authors also found significant varietal differences in response to these pests. In Thailand, Norsuwan et al. [58] reported that major insect pests of vegetable soybeans include bean flies (*Melanagromyza sojae*, *M. phaseoli*, *Ophiomia centrosematis*, *Dolichostigma* sp.), white flies (*Bemisia tabaci*), beet army worm (*Spodoptera litura*), leaf roller (*Lamprosema indicate*; *L. diamenalis*), stink bugs, bean pod borer (*Etiella zinckenella*), leafhoppers, and aphids. They also showed that application of yellow sticky trap can reduce infestations by bean fly (*Melanagromyza* sp.) and pod borer in vegetable soybeans by 68.5 and 51.6%, respectively, compared to control (no insect management).

#### 4.2.2. Diseases

Soybean rust, caused by *Phakopsora pachyrhizi*, is the most important foliar disease of soybeans worldwide. In China, vegetable soybean production is concentrated in the southeastern coastal area, where anthracnose frequently occurs because of the warm, humid climate [59]. In the mid-Atlantic U.S., Lord et al. [53] reported downy mildew, bacterial pustule, *Fusarium* pod rot, *Cercospora* blight, purple seed stain, and damping off as important diseases affecting vegetable soybeans. In India, the susceptibility of the varieties Swarna Vasundhara and Karune to YMD is a major constraint in the expansion of the crop to northern and central parts of the country. In Thailand, important diseases of vegetable soybeans include soybean mosaic virus and purple seed stain. The latter does not affect yield but reduces germination and seed quality.

### 4.3. Abiotic Factors

Insufficient rainfall or irrigation causes aborted blossoms, small pods, and shriveled beans in vegetable soybeans, thus greatly reducing the yield [60]. Moloi and van der Merwe [61] in their study of vegetable soybeans under controlled environmental conditions found that the tolerance responses of ascorbate peroxidase (APX), guaiacol peroxidase (GPX), and glutathione reductase (GR), (especially at the flowering stage), function in concert to minimize hydrogen peroxide (H_2_O_2_) production and lipid peroxidation, thereby allowing H_2_O_2_ to function in the signaling events leading to the induction of drought tolerance. The induction of total soluble sugars (TSS) at flowering and proline at pod filling was also important as a response to drought tolerance. AGS429 was the only variety that significantly increased H_2_O_2_ under drought stress.

### 4.4. Crop Management

Regardless of the progress that has been accomplished in the varietal improvement for better yield levels, poor crop management practices have constrained the successful and sustainable production of vegetable soybeans. Poor nodulation can hamper the nitrogen fixation ability of the plants which leads to lower yields. Planting depth directly affects the seedling emergence results in poor plant stands [4,62]. Close-spacing between plants leads to overly high densities in the field which triggers the competition for soil moisture and nutrients as well as for solar radiation [63] which ultimately results in poor plant growth and low productivity. Potassium deficiency in the soil can be detrimental to better crop growth and yield level [64]. Vegetable soybean growth and productivity are adversely affected by salt stress. It prolongs the bean filling duration, reduces the size of the bean, and ultimately affects yield potential [65]. Water logging stress due to excessive soil moisture in the field is detrimental to the crop at any growing stage. Increasing labor scarcity and lack of weedicide tolerant varieties [66] are the major barriers for better crop production. The highly perishable nature of vegetable soybean pods has narrowed down the harvesting window. This gives very few days to the farmers to harvest and sell the pods before they turn yellow and unmarketable in fresh markets [4,67]. Labor costs account for about 62% of the total crop production cost, most of which is for manual harvesting [68]. Mechanical harvesting can reduce about 28% of the total cost of production, but the mechanical damage while harvesting penalizes the yield up to 24% in comparison to hand harvesting [69]. In Taiwan, using a 190 HP, FMC 7100 harvester operating at 3400 rpm, the actual loss was kept around 5%, the damaged pods were around 7%, and the total cost of production was reduced by about 20% [70]. Dhaliwal and Williams [41] reported that the economically optimal plant density for machine-harvested vegetable soybeans ranged from 87,000 to 120,000 plants/ha. In the U.S., major obstacles for commercial production of vegetable soybeans are the efficiency of mechanical harvesting and the cost of hand harvesting, where manual harvesting is still a common practice for small farmer [53,71]. In Thailand, machine harvesting became popular for vegetable soybeans during the past few years as it greatly reduces the harvest cost.

### 4.5. Post-Harvest Practices

Pod quality and nutritional quality of vegetable soybeans are highly dependent on stage of harvest, storage conditions, and processing of pods [72]. Vegetable soybeans are more perishable due their high respiration rate after harvest, and subsequently, quality will decline. Based on consumer preference, soybeans can be marketed as fresh or frozen pods, beans, and canned products [2]. Cold storage includes refrigeration, and freezing is the best method to preserve vegetable soybeans without losing sensory attributes and nutritional qualities [73]. Fresh vegetable soybeans stored at 4 °C under a nitrogen atmosphere contained the highest sucrose content after storage. Water blanching of pods and steam blanching of pods or seeds at 100 °C for 10 min successfully preserved the sugar composition of vegetable soybeans. In order to minimize the quality losses, many farmers in Taiwan and in Thailand harvest the pods during the night. In the contract farming system, the process from harvest to transportation is less than 4 h, and the pods are stored in cold rooms during transportation from the farm to the company/factory.

Exposing pods or shelled beans to boiling water (blanching) before cold storage is an excellent method to improve shelf life. Blanching can inactivate enzyme activities and reduce microbial load but improve color, texture, and protein stability [74,75]. Xu et al. [76] recorded that blanching for 2.5 min or more at 100 °C in water reduced peroxidase activity by 98% and also increased the thermal denaturation temperature of vegetable soybean beans by 30 °C. Djanta et al. [29] emphasized the need for investigation on the optimized storage techniques that suit tropical environments of sub-Saharan African countries.

### 4.6. Quality Seeds

Good quality seed is essential for producing a good quality crop, and a number of factors, such as growing season, location, management inputs, diseases and insect pests, postharvest handling, and storage affect seed production. For seed production, locations with optimum temperature, low relative humidity, and dry weather during seed maturation time should be selected. The quality of the dry season crop is better than the rainy season crop. For example, in Japan, the vegetable soybean seed crop is produced in Hokkaido, where cool dry conditions and moderate temperatures help produce good quality seed. In Taiwan, the seed production is taken up mainly during the autumn season when the weather is cool and dry. [70]. Similarly, in Thailand, seed production sowing is done in the dry season from December to mid-January in northern Thailand. The major difference in cultural practices for the seed crop compared to grain soybeans is in pest and disease control. Intensive integrated pest and disease management measures should be undertaken to protect the quality of the mature seed. The pesticide residue is not a major issue as long as it does not affect the subsequent vegetable soybean crop. In locations where the day night temperatures are highly variable, most of the varieties will shatter upon maturity.

## 5. Constraints to Consumption

**Figure 2:**
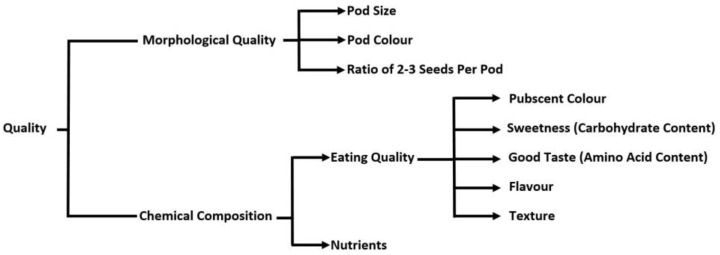
Key quality traits (Reproduced from [78]).

Pod quality (Figure 2) is paramount to fetch premium prices in the fresh or frozen market, particularly in Asian countries where consumers prefer to consume vegetable soybeans as a snack. Bright, green-colored pods are best, as yellowing indicates the depletion of nutritional quality (sugar is converted into starch, and the taste is unacceptable to consumers) and unsuitability for fresh markets [72]. Pod texture is also one of the major sensory attributes. For instance, glabrous pods (without hairs) feel better in the mouth when squeezing the boiled pod to extract the seed for a snack [77]. Pods with white or grey pubescence are preferred over those with brown or dark pubescence because of the clean appearance after cooking [1]. Three or more seeded pods are preferred for the fresh or frozen pod market. Fresh or frozen shelled beans are also marketed in most countries. In Japan, China, and Taiwan, pods not qualified for fresh use are processed for shelled beans. To attract a premium price, beans should be larger in size (about 50 to 60 g/100 beans), and color should be light green without any dark spots [1].

### Anti-Nutritional Factors

Trypsin inhibitor, a proteinaceous anti-nutritional factor present in soybean seeds and other legumes, affects protein digestibility. In both grain-type and vegetable soybeans, trypsin inhibitor (TI) has been reported to be present at the R6 stage [79], and Kunitz trypsin inhibitor (KTI) polypeptide is completely synthesized as the soybean plant reaches the R6 stage of the reproductive phase when the green pods are ready for harvest [80]. KTI is heat labile due to the presence of only 2-disulfide linkages; however, a minimum of 15–20 min moist heat treatment is required for its complete inactivation [80]. Although vegetable soybeans are boiled for 5 to 8 min prior to consumption, it is insufficient to completely inactivate the KTI in vegetable soybeans. TI is a problem more in grain soybeans than in vegetable soybeans, and therefore it does not deserve priority attention.

Taste, which is the important (Figure 2) determinant of organoleptic acceptance can be improved by enhancing the levels of sweetness-imparting soluble carbohydrate viz. maltose in the seed. Jha et al. [81] reported QTLs, namely, Sat_216 (LG-H/chr 12, 85.27 cm, LOD score = 3.18), Satt681 (LGC2/chr 6, 3.15 cm, LOD score = 2.54), and Satt720 (LG-E/chr 15, 20.8 cm, LOD score = 2.10) that are in the proximity of functional genes involved for the biosynthesis of enzymes, such as β-amylase, glycoside hydrolase, and UDP-glucosyl transferase which degrade starch into maltose. Allelic contribution of these genomic regions to maltose varied significantly and can be potentially useful in marker-assisted breeding for development of high maltose content soybean genotypes.

Both the sucrose-derived raffinose-family oligosaccharides (RFOs), i.e., raffinose and stachyose, are flatus-inducing biomolecules. Several studies have shown very low levels of raffinose and stachyose at the R6 stage during seed development in both grain type and vegetable soybeans [82,83]. This may be attributed to the absence of or very low concentration of raffinose synthase and stachyose synthase, which catalyze the synthesis of these molecules from sucrose, until the R6 stage of seed development, and thereafter spike when the moisture content starts declining, and the developing seeds approach maturity.

## 6. Technological Interventions to Address the Constraints

### 6.1. Varietal Improvement

#### 6.1.1. Improving Genetic Variability

**Figure 3:**
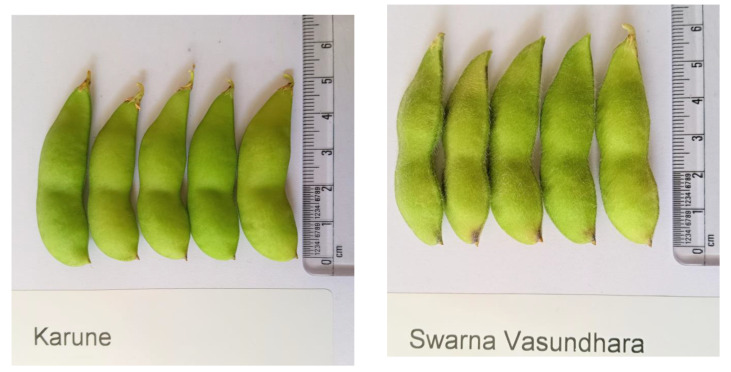
Karune variety with glabrous pod on the left and Swarana Vasundhara variety with pubescent pods.

**Figure 4:**
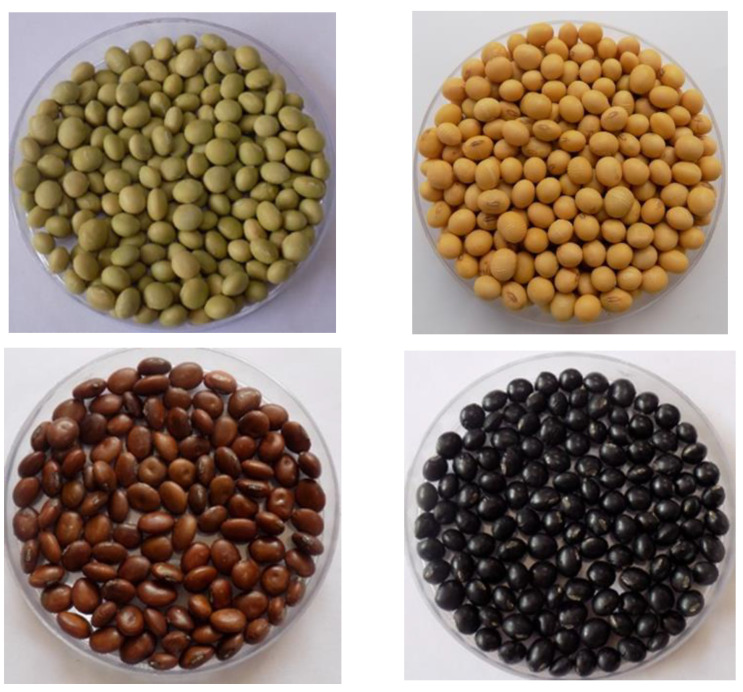
Variation in seed colour in vegetable soybean varieties.

Identifying new traits of interest in large germplasm collections is laborious and costly. Establishing subset of collections, either core collections, which represent the diversity of the whole collection, or subsets enriched for specific traits makes screening more practical [84,85]. Various soybean core and mini-core collections have been established, for example at the USDA in the USA [86], in China [87,88], in Korea [89], in Brazil [90], and in Japan [91]. A core collection (30 accessions) for Taiwanese vegetable soybeans have been described by Kao et al. [92]. Historical data (1973–2015) collected at the World Vegetable Center were utilized for developing soybean core collections representing the diversity of the whole collection of 7853 accessions held by the center [93]. The collection was split into two groups by Nair et al. [93] on the basis of the 100 seed weight: large seeded (> or equal to 25 g) and small seeded (<25 g). The large-seeded group (vegetable soybeans) comprised 456 accessions, while the small-seeded group contained 7397 accessions. Based on 7 quantitative (number of seeds per pod, plant height at R1 stage, plant height at R8 stage, number of primary branches, days to 50% flowering, number of pods per plant and 100-seed weight) and 14 qualitative (pubescence color, pubescence type (Figure 3), hypocotyl color, corolla color, mature pod color, seed color (Figure 4), seed coat pattern, hilum color, seed coat surface lustre, stem determination, leaflet shape, pubescence density, leaflet size, and lodging score) traits collected during the autumn season, a core collection of 111 large seeded vegetable soybean accessions and 1480 accessions for the small seeded types were developed. The core collection demonstrated a small percentage of significant mean difference (3.45%) and a large coincidence rate (97.70%), indicating representativeness of the entire collection. Furthermore, large values in variable rate (149.80%) and coverage (92.5%) were in line with high diversity retained in the core collection. The results suggested that phenotype-based core collections can retain diversity and genetic variability of vegetable soybeans, providing a basis for further research and breeding programs. The WorldVeg vegetable soybean core collection complements the vegetable soybean core collection of Kao et al. [92]. The WorldVeg collection is smaller and has a broader geographic range compared to the latter collection. In comparison to other soybean core collections, the WorldVeg collection contains a larger proportion of large-seeded vegetable soybean accessions. The definition of these core collections will help to enhance the use and management of the WorldVeg collection of soybeans [93].

Yu et al. [94] re-sequenced the four vegetable soybean varieties (Taiwan-75, Zhexiandou No. 8, Zhexian No. 9, and Zhexian No. 10) that are planted across large areas of Zhejiang province in China. Although the clustering of SSR analysis suggested the similar pedigree of these four varieties, they still showed a different distribution of genetic variations on each chromosome. The average heterozygosity rate of the single-nucleotide polymorphisms was 11.99% of these four varieties. According to the enrichment analysis, there were 23,371 genes identified with putative modifications, and a total of 282 genes were related to carbohydrate metabolic processes. These results provide useful information for genetic research and future breeding, which can facilitate the selection procedures in vegetable soybean breeding.

#### 6.1.2. Quality Traits

Seed size is an important visual quality trait and directly contributes to yield of vegetable soybeans. Extra-large seed varieties, for instance SX6 from China with a fresh 100-seed weight of 107 g (about 40% larger than a standard variety AGS292), were identified and suggested to be useful for breeding of new varieties of vegetable soybeans [18]. QTLs for 100-pod fresh weight, 100-seed fresh weight, 100-seed dry weight, and moisture content of fresh seeds at the R6 stage were identified through GWAS using 133 vegetable soybean accessions and 82,187 SNPs [95]. In total, 35 SNPs were repeatedly identified for these traits and mainly clustered with QTLs conferring yield-related traits. Markers AX-90496773 and AX-90460290 were associated with pod fresh weight and moisture content of fresh seeds, respectively. Based on gene expression analysis, genes *Glyma.16g018200*, *Glyma.16g018300*, *Glyma.05g243400*, *Glyma.05g244100* and *Glyma.05g245300* were regarded as candidate genes associated with the pod fresh weight and moisture content of fresh seeds. Moseley [96] reported the association of SNP ss715587475 at the 24,888,097 bp position, with seed weight and seed size traits, within the gene *Glyma.04G143300* in a study comparing the phenotypic and genetic diversity of seed weight and seed size traits among 343 large-seeded accessions from 7 different countries to 31 breeding lines from the University of Arkansas soybean breeding program. These candidate genes can be used to develop functional markers for breeding high-yielding vegetable soybeans.

Association mapping based on linkage disequilibrium involves searching for genotype–phenotype correlations in unrelated individuals [97]. The variation in quality traits, namely 100-pod weight, 100-seed weight, sucrose content, and free amino acid content in vegetable soybeans from China, were analyzed by Hou et al. [98], and SSR markers closely linked to these traits were identified by association mapping. A total of marker–trait associations related to the 4 traits were identified involving 44 markers. Association analysis could enable the quantitative trait loci mapping for marker-assisted selection. Sucrose is the main sugar component in vegetable soybean seeds, and its concentration is the most important determinant of vegetable soybean taste. Association analysis of vegetable soybean sucrose contents measured in 2 years (2008 and 2009) conducted using 323 Chinese vegetable soybean accessions and 101 SSR markers, revealed 9 markers associated with sucrose contents [98]. However, none of the SSRs were consistently associated with sucrose contents. QTL mapping in RILs developed by a cross G00-3213 × 594458A that evaluated seed sucrose contents in 4 environments revealed 10 QTLs on different chromosomes controlling the sucrose contents [99]. However, combined analysis identified five sucrose QTLs located on Chrs 3, 4, 6, 11, and 17. The Com_qSuc-1 (R2 = 10.6%) and Com_qSuc-3 (R2 = 13.6%) located on chromosomes 3 and 6, respectively, co-localized with the QTLs for the protein contents. At these QTLs, the allele effect for the two traits were large but opposite, suggesting that increasing seed sucrose content would be accompanied by decreased protein content. In another study, GWAS for sucrose content using 266 soybean accessions genotyping with 76k SNPs revealed QTLs on chromosomes 1, 6, 8, 9, 10, 13, and 14 controlling sucrose content. *Glyma.06g182700A* encoding carbonic anhydrase that has been shown to have an indirect role in the sucrose metabolic pathway was identified as the candidate gene for the QTL located on chromosome 6. These QTLs identification would be useful for designing a breeding program for high-sucrose content vegetable soybeans. Zhao et al. [100] investigated the inheritance of soluble sugar content in vegetable soybeans, and three QTLs were mapped on M, A2, and I linkage groups: explaining 6.89%;~14.54% of the total phenotypic variation, which could be potentially useful for marker-assisted selection.

A high-quality de novo genome assembly of the vegetable soybean cultivar Zhenong 6 (ZN6), one of the most popular varieties in China, was presented by Liu et al. [101]. The 20 pseudochromosomes cover 94.57% of the total 1.01 Gb assembly size, with contig N50 of 3.84 Mb and scaffold N50 of 48.41 Mb. A total of 55,517 protein-coding genes were annotated. The authors resequenced 60 vegetable soybean accessions, compared with previously resequenced 103 wild soybean and 155 grain soybean accessions. They found 1112 and 1047 genes under selection in vegetable soybean and grain soybean populations compared with the wild soybean population, respectively. Among them, they identified 134 selected genes shared between vegetable soybean and grain soybean populations. Four sucrose synthase genes, one sucrose–phosphate synthase gene, and four sugar transport genes as candidate genes were reported related to important traits, such as seed sweetness and seed size in vegetable soybeans.

KTI free grain-type soybean varieties have been developed in several countries [102,103,104]. For this purpose, the plant breeders deployed KTI null allele specific marker [105] and Ti linked SSR markers [106,107]. In India, genetic elimination of KTI from vegetable soybeans was developed by crossing Dadachamame and NRC105, the two vegetable-type genotypes, as the recipient parents with NRC101 [108]. NRC101 is the first KTI free grain-type genotype developed through marker-assisted selection [109]. Maintaining high sucrose content and seed size, the two major characteristics of vegetable soybean genotypes, while removing KTI was a challenge. Large F_2_ populations segregating for null allele KTI were screened using null allele specific markers to identify KTI null plants. Both homozygous recessive (titi) and heterozygous plants (Titi) were advanced to the F_6_ generation. At this advanced generation, large-seeded lines (>50 g: 100 fresh green seed weight) were analyzed for null allele specific marker in tandem with linked SSR marker Satt538 to identify homozygous recessive (titi) plants. These were confirmed through native PAGE and Western blot for the absence of KTI. All KTI-free (titi) with large seed size were screened to identify high sucrose content (>7%) lines at the R6 stage and morphologically similar to the recipient parents. This led to the development of novel vegetable soybean lines free from KTI [110].

Efforts are in progress for marker-assisted elimination of lipoxygenase-2 gene from vegetable soybeans. Null allele of lipoxygenase-2 gene is being introgressed in vegetable soybean genotypes NRC105 and Karune, using ‘NRC109′ -null lipoxygenase-2 Indian soybean genotype- as the donor parent, through marker-assisted backcrossing deploying lipoxygenase-2 null allele specific marker and Lox2 linked Satt656 marker reported from an earlier study [111].

The special fragrance in vegetable soybeans is due to the presence of 2-acetyl-1-pyrroline (2AP), the same major volatile chemical that confers fragrance even in other crops including rice. Genetic mapping [112] using an RIL population developed from a cross between a fragrant line, Kaori, native to Japan, and a non-fragrant line, Chiang Mai 60, uncovered that the QTL position of fragrance and 2AP production coincides with the position of *GmBADH2* (*Glycine max* betaine aldehyde dehydrogenase) gene. A single nucleotide polymorphism (SNP) in the *GmBADH2* gene was found associated with the fragrance in Kaori. The authors developed PCR-based SNP markers for utilization in breeding programs. This was recently confirmed by Qian et al. [113] in three novel aromatic vegetable soybean varieties (QX1, ZK1754, and XD). The pandan-like fragrant variety Chiang Mai 84-2 has been developed to replace the variety Kaori for export to Japan [43].

At WorldVeg, GC-84501-32-1, a line identified with high seed viability [114] is being used to improve seed viability of commercial varieties.

#### 6.1.3. Resistance to Insect Pests and Nematodes

Soybean pod borer (*Leguminivora glycinivorella* Matsumura) is a major insect pest in vegetable soybean production. Soybeans with glabrous pods appear to be resistant to the soybean pod borer [115]. Glabrous pod also contributes to eating quality. Gene controlling glabrous pod was mapped in an F_2_ segregating population derived from the cross between vegetable soybean lines “Aijiaomaodou” and “39,002”. The results showed that glabrous phenotype was controlled by a single dominant gene. Gene mapping using genome-wide SNPs markers generated by polymorphic specific-locus amplified fragments identified the 2.76-Mb genome region (47,315,610–50,072,564) on chromosome 9 containing 551 candidate genes associated with the glabrous trait. Four genes including *Glyma09G280500*, *Glyma09G282600*, *Glyma09G259400*, and *Glyma09G283400* in the region appeared to be the most important candidate genes that are highly related to the glabrous trait [116].

A survey of nematode genera and density in 64 contracted vegetable soybean production fields in Arkansas, USA was conducted during 2013 and 2014. In both years, *Meloidogyne* and *Heterodera* were present in less than half of the surveyed fields, while *Pratylenchus* was the most prevalent in the year 2013 and *Helicotylenchus* in 2014. Four lines showed consistent reduction in M. incognita reproduction relative to the commercial varieties and could represent sources of moderate resistance for development of future root-knot nematode resistant vegetable soybean varieties [117].

#### 6.1.4. Resistance to Diseases

Soybean rust can cause up to 100 per cent yield loss in vegetable soybeans. Murithi et al. [118] identified vegetable soybean lines: AGS 339, AGS 423, AGS 459, and AGS 461 with rust resistance in Mikumi region of Tanzania. Zhu et al. [59] developed a method for inoculating pods in vitro by soaking in a mycelial suspension to evaluate anthracnose resistance. The authors optimized the crucial components, including the mycelial suspension concentration (40 to 60 mg mL^−1^), the maturity of the sampled pods (15 days after flowering), and the post-inoculation incubation period (5 days). Resistant lines identified will pave the way for gene discovery, elucidation of molecular mechanisms, and the breeding of resistant varieties. One of the main challenges faced in the expansion of the crop in India was the susceptibility of vegetable soybean varieties to yellow mosaic disease (YMD), caused by *mungbean yellow mosaic India virus* (MYMIV). Khosla et al. [119] found that out of 267 SSR markers, 114 markers showed parental polymorphism in a bulked segregant analysis (BSA) of F_2_ population derived from cross SL 958 (resistant) × AGS 456 (susceptible). Composite interval mapping revealed one major QTL on chromosome 6 flanked by markers Satt281 and Sat_076 and one on chromosome 2 flanked by SSR markers BARCSOYSSR_02_0423 and BARCSOYSSR_02_0425. Furthermore, gene annotation with these QTLs on soybean genome also located two candidate genes, namely, *RDRP1* and *SGS3* in these regions having a role in resistance against begomoviruses. Markers identified in this study will be helpful in marker-assisted breeding for the trait. Improved lines resistance to the disease were developed by WorldVeg by utilizing SL 958 as the source of resistance (https://avrdc.org/seed/improved-lines/vegetable-soybean/ (accessed on 1 February 2022)).

#### 6.1.5. Maturity

Days to flowering was studied utilizing an RIL (recombinant inbred line) population of 92 lines derived from the cross between early flowering line AGS292 and late flowering grain soybean line K3. The RILs were analyzed with 63 SSR markers for QTL analysis. The QTL analysis revealed the markers Satt431 on linkage group J showed the strongest association with days to flowering (R2 = 27%). This marker can be potentially useful for selection of early or late flowering genotypes during the seedling stage [120].

### 6.2. Good Agricultural Practices

Good agricultural practices (GAP) assure a significant effect on quality and yield of vegetable soybeans. Seed treatment with bradyrhizobia (*Bradyrhizobium japonicum*) inoculant (10 g per 1 kg seed) enhances the root nodulation to improve nitrogen fixation [7,121]. Planting depth is a crucial aspect to increase the seedling emergence in the field. Sowing the seeds at 1–3 cm depth in soil can significantly increases the emergence than a deeper (5 cm) planting depth [62]. However, sowing with shallow planting depth may result in poor germination due to insufficient soil moisture. Plastic mulching is also being practiced for better weed control. Staggered planting of seeds in different blocks can extend the harvest window to avoid post-harvest losses and enable a steady supply to the market [67]. For an extensive review on various factors related to GAP including organic vegetable soybean production, see [1]. Good seedling emergence and optimum plant population are key to commercial production of the crop. Sanchez et al. [122] in their field trials conducted in Central Pennsylvania in the USA during 2002 to 2004, found that the plant populations varied with the year and variety and was below 80%. Under controlled conditions, the authors found that when grown in a 70/60 °F day/night temperature regime, varieties ‘Butterbeans’ and ‘Early Hakucho’ exceeded 80% seedling emergence. In their work on vegetable soybeans in the Arkansas and the United States Mid-South, Moseley et al. [123] suggested an Edamame Harvest Quality Index combining phenological stages, thermal units, and planting dates. Their results indicated that the vegetable soybean quality is increased with delayed planting dates and that quality was dependent on harvest date with a quadratic negative response to delaying harvest. Maximum quality depended on variety and planting and harvest dates, but it remained stable for an interval of 18–27 days around the peak. The authors also observed that the number of days between the R1 stage and harvest was consistently identified as a key factor driving vegetable soybean quality by both stepwise regression and neural network analysis. In South Africa, Arathoon [124] worked on the most suitable seeding rate, fertilizer requirement, the effect of seed coatings with fungicides, and *Bradyrhizobium japonicum* Kirchner inoculants on plant population, nodulation, and yield and estimated the costs and profitability of producing and marketing the crop in KwaZulu-Natal.

Restaurants normally prefer fresh vegetable soybeans. Owing to their highly perishable nature, maintaining the post-harvest shelf-life and minimizing the physicochemical and microbial deterioration that causes degradation of their color, texture, and flavor are critical. In studies conducted in Brazil, Santana et al. [125] reported that in order to preserve the quality of vegetable-type soybeans, pods should be stored at 30 °C and consumed within 24 h or stored at 7 °C for up to 3 days of storage. Kim et al. [126] in their investigations found that a combination of steam blanching for 30 s at 90 °C, vacuum packaging and cold storage at 4°C helped to prolong the physical and microbiological quality of vegetable soybeans. The potential of infrared heating as a processing methodology to dry and blanch vegetable soybeans was demonstrated by Lara et al. [127].

### 6.3. Quality Seeds

Considering the nutritional importance and health benefits in addition to economic benefits of vegetable soybean cultivation, particularly under rainfed upland ecosystems, the demand for vegetable soybeans is sure to increase throughout the world [51]. Production of quality seed and its supply to the farmers/growers in time would make the vegetable soybean production a successful venture both at home garden scale as well as commercial scale. In China, most of the vegetable soybean varieties were developed by governmental institutions. In Japan, most of the varieties have been developed by seed companies. However, some local varieties with special flavor are grown locally. In Taiwan, farmers grow vegetable soybeans which are all bred by Kaohsiung DARES (100%). In Thailand, all the vegetable soybean varieties used for export are exotic and promoted by export companies. The companies produced 30–40 of the seeds used and imported the rest from Taiwan. Some varieties for export and domestic use are developed by governmental institutions. The institutes also produced the commercial seeds but with limited volumes. A few varieties for domestic consumption are also developed by farmers through reselection of grain-type soybean cultivars. In the latter case, the farmers save the seeds for their own use (growing and selling).

The WorldVeg South Asia in cooperation with the ICAR Research Complex for the Eastern Region Farming System Research Center for the Hill and Plateau Region promoted the improved vegetable soybean variety, Swarna Vasundhara, particularly among tribal farmers of Jharkhand state of India during 2008–2012. This effort showed that seed production of vegetable soybeans through farmers’ cooperatives could help supply seeds to interested farmers [3]. As production and supply of quality seeds is of major concern for the expansion of vegetable soybean production areas, large-scale seed production of vegetable soybean needs to be undertaken by the respective national seed production systems of concerned countries through seed village projects with an assured seed buyback policy for seed producers. The involvement of NGOs/private companies for seed production programmes and supplying quality seeds of vegetable soybeans to farmers is another approach to be tried as it was successful for other vegetable varieties including soybeans of the World Vegetable Center in Kenya, Bangladesh, India, Uganda, and Tanzania (World Vegetable Center, 2019). In this type of programme, a group of selected farmers is chosen and properly trained on quality seed production techniques so that a minimum standard and germination percentage of produced seeds are maintained. The national agricultural research institutes/state agricultural universities can also take up farmers’ participatory seed production programmes of vegetable soybeans under direct supervision of scientists of the research institutes. For example, in the first year of the World Vegetable Center South Asia project, “Improving Vegetable Production and Consumption for Sustainable Rural Livelihoods in Jharkhand” sponsored by the Sir Ratan Tata Trust during 2008–2009, the seeds of Swarna Vasundhara vegetable soybean variety produced through the farmers’ participatory approach were supplied from the ICAR Research Complex for Eastern Region Farming System Research Centre located at Ranchi (23°15′ & 23°18′ North and 85°20′ & 85°25′ East; Elevation 620 m above mean sea level) in Jharkhand state of India [128]. The farmer’s/seed producers can grow successful seed crops of vegetable soybeans adopting the same cultural practices followed in the case of crops for green pod harvest [129]. For early maturing varieties, the seed crop can be sown in July so that weather remains dry during dry pod and threshing, and the drying of seeds becomes easier. Being a self-pollinated crop, the isolation distance for foundation and certified seed production should be 50 m and 25 m, respectively. When the plants mature, the leaves turn yellow and senesce. The pods are dried, and the seeds lose considerable moisture. At harvest, the moisture content of seeds should be 15–17%. The maturity period ranges for 90–120 days, depending on the variety. The whole plants with dried pods should be cut at soil level and taken to the threshing floor. As seed shattering is a problem, harvesting should be performed in the morning hours. For threshing, optimum moisture content with least damage to the seeds is 18 to 20% (in Japan it is 16 to 18%), and the cylinder speed of the threshing machine should be adjusted to 10 to 11 rpm/s [130]. Following threshing, the seed should be dried at 30 to 35 °C for 24 to 48 h. The seed should be dried to bring the seed moisture content down to about 9–10%. A simple practical guide for seed moisture content is as follows: If the seed readily splits and cracks (when crushed), then the moisture content is about 10%; when the seeds are crushed on the drying floor and the floor becomes sticky, the moisture content is more than 10%. The following are the guidelines for vegetable soybean seed quality: (1) 98% purity, (2) other species seeds less than 0.2%, (3) inert matter less than 2%, and (4) germination should be more than 85%. The dried seeds can be stored in 0.03 mm thick polyethylene bags, sealed airtight and stored in a cool, dry place [130]. The seed yield of vegetable soybeans ranges from 0.9 to 1.2 t/ha. Vegetable soybean seeds can also be stored in metal bins by adding ash to prevent moisture fluctuations. The metal bins are sealed airtight with a lid with a rubber gasket. Vegetable soybean seeds can also be stored in air-conditioned rooms at 25 °C and 65% relative humidity. Under the above conditions, seeds with 10 to 11% moisture content retain their initial germination rate and seed vigor for one year in storage. At the World Vegetable Center, seeds with 8 to 10% moisture content stored in airtight containers or hermitically sealed in high-density polyethylene bags could maintain high seed viability for two years at 30 °C [70].

James [49] reported that seed markets for vegetable soybeans are well developed in countries where vegetable soybeans are adopted and well consumed. Many private and public organizations have great interest in the crop, and seeds of superior varieties are nicely packed and marketed. In the Netherlands, “DutchSoy” representing the “Europe Soya” organization is producing and promoting organic vegetable soybeans with many soybean varieties. In addition to selling seeds, the company provides farmers with technical assistance. The firm conducts field trials and research to develop technologies, to supply quality seeds to producers, and to provide them with advice on various aspects of non-genetically modified (GM) vegetable soybean production. It also assists them with market strategies and distributes rhizobia to them.

In Southeast Asian countries such as Indonesia, Thailand, and Vietnam, and in South Africa, well-developed seed production and distribution systems with backstopping for vegetable soybean production, processing and marketing as well as promotion of vegetable soybeans to consumers are available [1,46]. In Brazil, the first vegetable soybean variety, BRS 267 soybean, was recently released. Embrapa is assisting farmers in the production technology and organizing the seed supply chain for the market, aiming to expand supply (https://www.embrapa.br/en/busca-de-noticias/-/noticia/60157325/first-brazilian-soybean-developed-for-consumption-as-edamame-a-popular-food-in-the-east (accessed on 3 February 2022)). The promotion efforts of vegetable soybean production should be well aligned with a sustainable seed production system [29]. It is critical to have both the formal and informal systems of seed production encouraged to meet the demands of the growers.

### 6.4. Potential Means to Enhance Consumer Demand and Consumption

Among the transgenic crops, grain soybeans tolerant to glyphosate herbicide, or Roundup Ready^®^ (RR) soybeans, stand out due to their particularly high expansion level and the extent of the area they cover [131]. However, the vegetable soybean breeding programs have traditionally maintained a non-GM approach, owing to its primary use in the food industry and also as a niche high-value product. For example, in the USA, consumers are willing to pay a price premium for non-GM vegetable soybeans [132]. Neill and Morgan [133] in their analysis of vegetable soybeans in the USA point out that the crop has great potential but incentivizing farmers to grow the crop requires a deep understanding of the added economic risks. Greater awareness about the nutritional importance of vegetable soybeans and their difference from grain soybeans, particularly the way they are consumed, is paramount. Promotion activities should include preparation of recipes acceptable to the local communities. Consumer preferences have to be assessed in order to develop suitable vegetable soybean varieties for sub-Saharan African countries [29].

## 7. Future Outlook

The demand for vegetable soybeans is slowly increasing. An awareness has been created by the World Vegetable Center in a large number of countries around the world for vegetable soybeans. New countries have commenced commercial production of vegetable soybeans both for domestic consumption and for export. The export market is rather finite and limited to Japan, the USA, the UK, Europe, Hong Kong, and Singapore. Most of the newly producing countries are looking for opportunities to export rather than promote and use for domestic consumption. Efforts should be made to improve the domestic consumption of vegetable soybeans in various countries. It is vital for the vegetable soybean breeding programs to have well-defined product profiles developed in consultation with the major stakeholders so that the improved varieties are well accepted by the consumers and the adoption rate will be enhanced. In parallel, the availability of the seed of the improved varieties should be ensured through both formal and informal seed systems.

## Figures and Tables

**Table 1:** Vegetable soybean area and production in selected countries (Updated from [1]).

Country	Area (ha)	Productions (t)	Year	References
China	400,000	2,000,000	2016	[7]
Japan	13,000	66,100	2019	[32]
Taiwan	9180	84,490	2018	[31]
Thailand	4000	25,000	2022	[33]
Indonesia	1417	11,202	2017	[34]

## Data Availability

Not applicable.
